# Diffdigester.uni-jena.de: a tool for optimized selection of restriction enzymes for plasmid identification in cloning procedures

**DOI:** 10.1093/nar/gkaf418

**Published:** 2025-06-04

**Authors:** Martin Gühmann, Stefanie Reuter, Ralf Mrowka

**Affiliations:** School of Biological Sciences, Faculty of Life Sciences, University of Bristol, 24 Tyndall Avenue, Bristol BS8 1TQ, United Kingdom; Experimentelle Nephrologie, Un iversitätsklinikum Jena KIM III, Am Nonnenplan 4, D-07743Jena, Germany; ThIMEDOP, Universitätsklinikum Jena, Am Nonnenplan 4, D-07743Jena, Germany; Experimentelle Nephrologie, Un iversitätsklinikum Jena KIM III, Am Nonnenplan 4, D-07743Jena, Germany; ThIMEDOP, Universitätsklinikum Jena, Am Nonnenplan 4, D-07743Jena, Germany

## Abstract

Differential digests, also known as test or diagnostic digests, are a standard method in molecular cloning to verify whether a picked clone is indeed the target plasmid or not. However, finding the optimal restriction enzyme for a differential digest by hand may be challenging and time-consuming. To address this problem, we created diffdigester.uni-jena.de (https://diffdigester.uni-jena.de), a free online tool to easily find such enzymes. This tool uses regular expressions to find the restriction sites in the DNA sequences given by the user. It then calculates and displays the resulting fragments on a simulated gel for each enzyme, allowing for easy comparison between the different enzymes. The user can sort these gels alphabetically by enzyme name or by dissimilarity of the restriction patterns between the given DNA sequences. This way, the most distinct pattern is shown first, which gives the most useful enzyme to distinguish between wanted and unwanted ligation products. In fact, it also works on completely unrelated sequences, expanding its possible applications. Thus, diffdigester.uni-jena.de is a fast, reliable, and free-to-use tool to help researchers plan differential digests for verifying their ligation products.

## Graphical Abstract

**Graphical Abstract:**
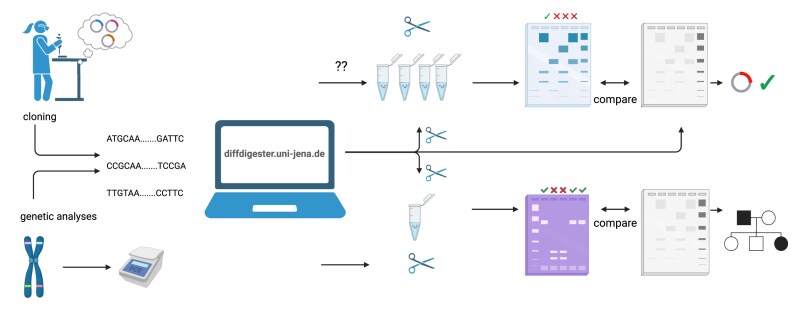

## Introduction

**Figure 1.**
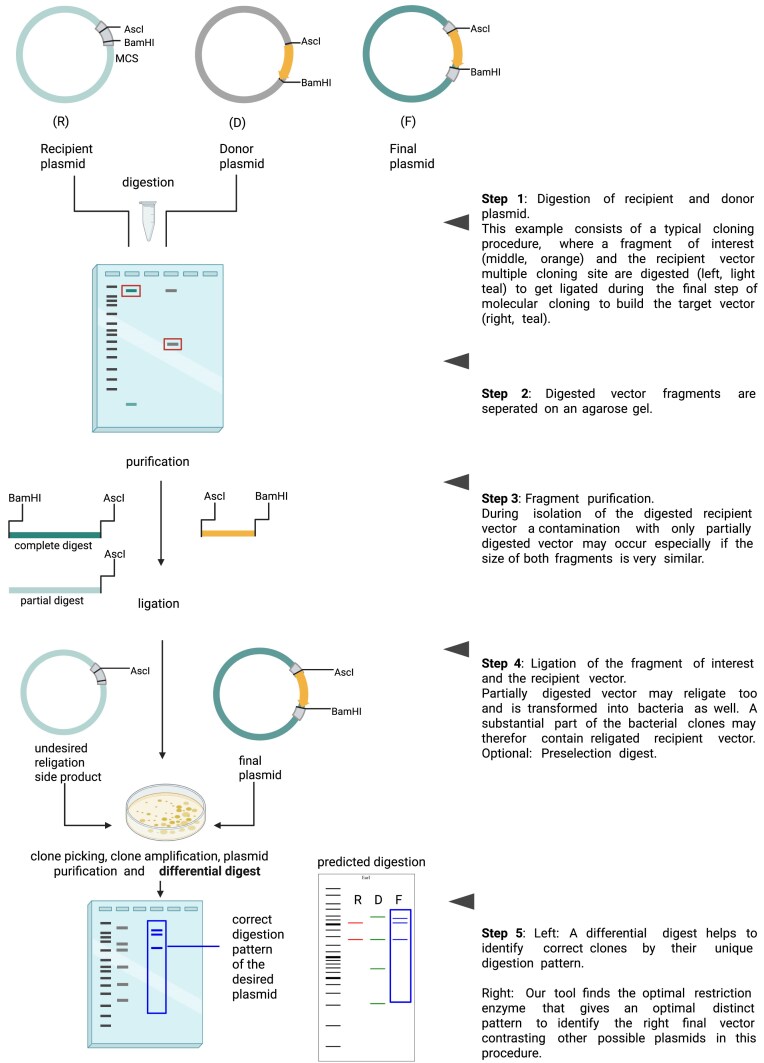
A typical procedure in molecular cloning that ends with a differential digest to identify a clone by the restriction pattern with a suitable enzyme. Diffdigester.uni-jena.de finds such enzymes for testing your clones. Created in BioRender. Reuter, S. (2025) https://BioRender.com/r56y919.

Molecular cloning is a standard method in molecular biology to combine DNA fragments from different sources into a circular plasmid. It is used, for example, for producing proteins in high amount, such as insulin [1], for genome editing with CRISPR–Cas9 [2], or for developing and producing nucleotide-based vaccines [3, 4]. For molecular cloning, restriction enzymes can be used to cut out a target fragment from a donor plasmid or a polymerase chain reaction (PCR) product. The target fragment is then ligated into a recipient plasmid that was also opened with restriction enzymes before [5]. Typically, the DNA fragments are purified and separated on an agarose gel before ligation (Fig. 1), which is, however, a source of contamination.

The restriction enzymes may digest incompletely and leave, for instance, a linearized plasmid of 10 000 bp, which is difficult to separate from a backbone that is just a few 100 bp shorter, as this is the case with cutouts from multiple cloning sites. Even if the fraction of linearized plasmid is low, it is easily religated and competes with the target final plasmid. There are also cloning strategies where the insert can be inserted in both directions, be it because the overhangs are compatible for that or because it is blunt-end ligation.

In the simplest case, the picked clones can have two identities: the recipient plasmid and the target plasmid. If the insert can be inserted in both directions, than there is another identity with a reverse insert. A clone can be identified by a differential digest, also known as a test or diagnostic digest. Here the restriction pattern shows which of the possible ligation products the clone is (Fig. 1). Differential digests are also used in non-traditional cloning methods such as Gibson assembly [6–8]. However, finding the optimal restriction enzyme by hand is not trivial and time-consuming and may lead to errors, potentially costing more time and work.

There are many programs that can help with designing cloning strategies, such as MacVector (https://macvector.com/), SnapGene (https://www.snapgene.com/), SeqBuilderPro (https://www.dnastar.com/software/lasergene/seqbuilder-pro/), Benchling (https://www.benchling.com/), Geneious (https://www.geneious.com/), and ApE (A plasmid Editor) [9]. These programs have many features, such as automatically annotating features in DNA sequences, DNA translation, designing PCR primers, and simulating restriction digests on agarose gels [9]. Gel simulation is needed to find the optimal enzyme for a differential digest. Additionally, the gels must be scored and compared automatically, to identify and select the optimal restriction enzyme based on dissimilarity between the different restriction patterns. This is a feature these programs do not have, and the user is left to compare such gels by hand.

To close this gap, we introduce diffdigester.uni-jena.de, a free tool to automate this tedious task. There, the user enters two or three DNA sequences and selects enzymes from a list. For each suitable enzyme, the site simulates a restriction pattern on a gel. By default, the gels are sorted with the most distinct pattern appearing first. The sorting is based on the shortest path root measure [10], a mathematically defined dissimilarity measure. Diffdigester.uni-jena.de processes everything in the local browser so that no sequences are uploaded to the server. This speeds up the workflow and allows quite long sequences, and the sequences stay confident, unlike it is the case with other online tools.

## Materials and methods

### Programming

For diffdigester.uni-jena.de, we reused in part code from our previous web server tool, preselector.uni-jena.de [11], for sequence loading and restriction enzyme handling. The restriction enzymes and their recognition sites are loaded from a database file that is derived from REBASE [12] and only contains commercially available enzymes. On top, we implemented everything that makes diffdigester.uni-jena.de, the logic for determining the number of fragments, displaying the gels, and sorting them.

Everything is implemented in JavaScript, which runs in the local browser. Therefore, no data are uploaded to the server, which saves time and all sequences stay confidential on the user’s computer. To find the restriction enzyme cut sites, we used regular expressions from standard JavaScript. Before matching, the input sequences and the recognition sequences of the enzymes are converted to lowercase for case-insensitive comparison. For each enzyme, the recognition sequence and its reverse complement are used to find recognition sites on the plus and minus strands. Any ambiguous base in a recognition sequence is translated into a regular expression following the IUPAC base pair definitions. When all the cut positions of an enzyme are found, the positions are used to calculate the fragment lengths, which are then drawn as bands on a simulated gel. The gels are drawn as vector graphics on the browser canvas. For circular matching, we concatenate the sequence from the last match to the end of the sequence with the start of the sequence until the first match and search for restriction sites within this combined piece. For simplicity, our simulated gels do not distinguish between linear, circular, and circular supercoiled sequences.

The gels are generated for each restriction enzyme and the user can sort them alphabetically by restriction enzyme name or by dissimilarity. To measure the dissimilarity between the band patterns, we used the shortest path root measure [6], a mathematically defined dissimilarity measure.

### Molecular cloning procedures

We planned all molecular cloning procedures *in silico* with Vector NTI 9.1 (Invitrogen) and designed the differential digests with diffdigester.uni-jena.de (https://diffdigester.uni-jena.de/).

#### Vectors and sequences used for cloning

The vector pcDNA™5/FRT (Cat. V601020) was bought from Invitrogen™ (Thermo Fisher Scientific). The vector pcDNA3-EGFP was a gift from Doug Golenbock (Addgene plasmid # 13031; http://n2t.net/addgene:13031; RRID:Addgene_13031). The vector pGuide-it-tdTomato (Cat. 632604) was bought from Clontech Laboratories, Inc. and pcDNA3-CD14 was a gift from Doug Golenbock (Addgene plasmid # 13645; http://n2t.net/addgene:13645; RRID:Addgene_13645). All sequences are available at https://diffdigester.uni-jena.de/.

#### Example 1: replacing a fluorophore gene by another gene of interest and an oligo adapter

1.2 μg recipient plasmid pcDNA™5/FRT (Invitrogen) was digested in a final volume of 25 μl with 20 units of both enzymes XhoI and PspOMI (NEB) in the provided rCutsmart buffer for 4 h at 37°C. The digested vector was run on a 1% agarose gel and the vector backbone containing band was purified from it by NucleoSpin Gel and PCR Clean-up Kit (Macherey-Nagel). The insert donor plasmid pcDNA3 containing human CD14 was digested in a final volume of 25 μl with 20 units of both enzymes HindIII-HF and NotI-HF (NEB) in the rCutsmart buffer for 4 h at 37°C. The digested vector was run on a 1% agarose gel and the insert band was purified from it by NucleoSpin Gel and PCR Clean-up Kit (Macherey-Nagel). The adapter oligonucleotide bridge incompatibles were diluted to a final concentration of 100 μM and mixed with each other in equal amounts. Fifty nanograms of digested recipient plasmid, 2 μl of mixed adapter oligos, and a three-fold molecular excess of insert DNA were ligated by T4 ligase (NEB) according to the manufacturer’s protocol. The ligated plasmids were transformed into top 10 chemically competent *Escherichia coli* by incubating bacteria and ligation products for 5 min on ice and plating onto LB agar medium (100 μg/ml ampicillin). Petri dishes with plated bacteria were grown overnight at 37°C. Single clones were picked and grown overnight in 5 ml of LB medium (100 μg/ml ampicillin) and plasmid isolation was performed using the NucleoSpin Plasmid Kit (Macherey-Nagel). 1.2 μg plasmid was used for the differential digests with 10 units of NcoI for 4 h at 37°C.

#### Example 2: replacing a fluorophore gene by another fluorophore gene amplified by PCR

1.2 μg recipient plasmid pcDNA3-CMV-EGFP was digested in a final volume of 25 μl with 20 units of both enzymes XhoI and XbaI (NEB) in the provided rCutsmart buffer for 4 h at 37°C. The digested vector was run on a 1% agarose gel and the vector backbone containing band was purified from it by NucleoSpin Gel and PCR Clean-up Kit (Macherey-Nagel). The Td tomato encoding fluorophore gene was amplified by PCR from pGuide-it-tdTomato (Takara, Clontech) using the following oligonucleotides: tomato-forward-XhoI gtacctcgagCCTACTTGTACAGCTCGTCCATGCC and tomato-reverse-XbaI cgtatctagaATTCGCCACCATGGTGAGCAAGG. The PCR product of about 1400 bp was purified after agarose gel electrophoresis and digested with the same enzymes as for the recipient vector. Fifty nanograms of digested recipient plasmid and a three-fold molecular excess of insert DNA were ligated by T4 ligase (NEB) according to the manufacturer’s protocol. Transformation, clone picking, and differential digests with AvaI (NEB) were done as described above.

#### Example 3: cloning a guide oligonucleotide into a Cas9 encoding plasmid

Forward and reverse DNA oligonucleotides for encoding a small guide RNA targeting the human IL-6 gene (forward: ccggGTGGAGAAGGAGTTCATAGC; reverse: aaacGCTATGAACTCCTTCTCCAC) were custom synthesized by MWG Eurofins and diluted to a final concentration of 100 μM. They were ligated into pGuide-it-tdTomato (Takara, Clontech) following the manufacturer’s protocol. Transformation, clone picking, and differential digests with XmnI (NEB) were done as described above.

### Graphics

The graphics were drawn with Biorender.com.

## Results and discussion

To solve the problem of finding the optimal enzyme for a differential digest, we set up the interactive website diffdigester.uni-jena.de. There, the user pastes or loads from a file two or three sequences, either plain or in FASTA format. The user may also specify whether the sequences are linear or circular.

Additionally, the user has several options to customize the search:

- Minimum and maximum fragment numbers, for which we set 2 and 10 as default values, respectively. Enzymes producing fewer than two fragments may not be useful for differential digestion as this is not really a pattern. And having too many fragments results in patterns that are less distinguishable.
- Minimum and maximum fragment length, for which we set 50 and 10 000 bp as default values, respectively.
- A cutoff fragment length, to cut off the gels at the bottom, which we set to 50 bp as default value. Primarily, this is for technical reasons, as we use a logarithmic scale for the band sizes to simulate real gels. However, a logarithmic scale means some empty space at the bottom of the gel, which we cut off with this setting. In fact, it also matches the situation in real-life gel electrophoresis, as resolving the bands of long fragments may require running the gel so long that the smaller fragments may even run out of the gel.
- Sort order of the gels: Here, the user can select whether the gels should be displayed alphabetically by enzyme name or by dissimilarity of the restriction patterns between the given DNA sequences. For the sort order “by dissimilarity”, we use the shortest path root measure [6], a mathematically defined dissimilarity measure. These sort orders can be reversed.
- The enzymes to use: The user selects them from a list and can save them to a file for later reuse. This allows easily repeating previous searches or sharing the enzymes with others.

Once everything is in place, the user only needs to press the “Submit and Digest” button and the website will simulate for the given sequences a digesting pattern for each enzyme. Each gel includes a DNA ladder with annotated sizes in lane 1. Lanes 2–4 contain the digestion pattern for each sequence in different colors. All these steps are illustrated in a Quick Guide on diffdigester.uni-jena.de in the Documentation and Help tab, so that the user can quickly look them up.

**Figure 2.**
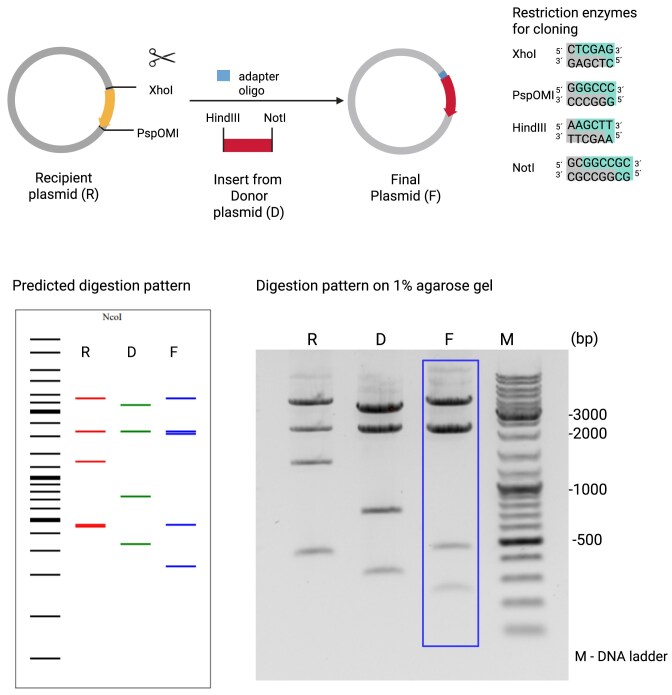
Replacing a fluorophore gene encoding mTFP by a T2A-CD14 cassette and an oligo adapter. In this example, a fluorophore gene encoding mTFP is replaced by a T2A-CD14 (glycoprotein recognition protein) cassette and an adapter oligonucleotide to bridge the non-compatible restriction enzymes XhoI and HindIII. The recipient and the donor plasmid are digested with two different combinations of restriction enzymes to generate fragments for the ligation. PspOMI and NotI generate compatible overhangs, which is not true for XhoI and HindIII. For that reason, an adapter oligonucleotide with compatible overhangs for both enzymes was added during ligation to get the final plasmid. Our tool suggests the enzyme NcoI to create digestion patterns of all three plasmids (R, D, and F), which are easily distinguishable. In comparison to the predicted digestion pattern, the agarose gel image is displayed. Created in BioRender. Reuter, S. (2025) https://BioRender.com/xuouvxu.

**Figure 3.**
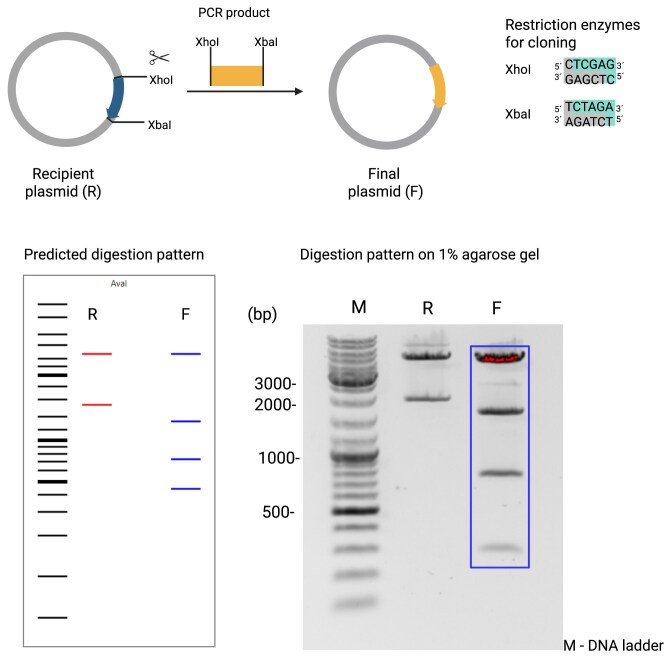
Replacing the sequence eGFP by that of tdTomato, another fluorescent protein, amplified by PCR. The recipient plasmid, encoding eGFP, and the PCR product of the fluorophore tdTomato are digested by the restriction enzymes XhoI and XbaI. The ligation of both fragments results in the final tdTomato encoding plasmid. A digestion of isolated bacterial clones with AvaI allows us to identify the correct clones by their digestion pattern. Created in BioRender. Reuter, S. (2025) https://BioRender.com/ilckde7.

**Figure 4.**
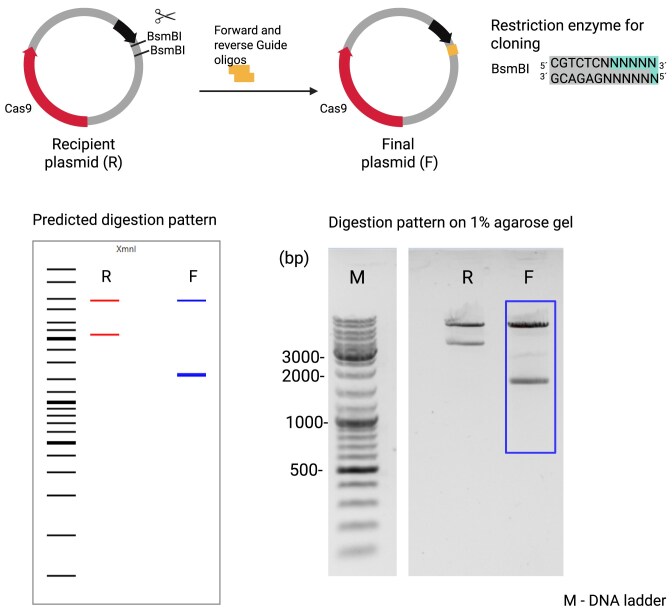
Cloning a guide oligonucleotide into a Cas9 encoding plasmid. CRISPR–Cas9-dependent gene editing is a standard method in genetic engineering approaches. Here, we used a backbone containing a fluorescent-tagged Cas9 with a single guide RNA (sgRNA), which can be used to guide the Cas9 enzyme to a specific location on the genome to induce a double-stranded break, in this case at the human IL6 gene. This plasmid is required for the first step for the modification of that particular location on the genome. The next step would require additional plasmids containing desired modifications with homologous regions in the vicinity of the double-strand break (not shown). Guide oligonucleotides for Cas9 applications are usually short, between 20 and 25 nt in length; therefore, it might be difficult to find a suitable enzyme and digestion pattern to distinguish between recipient and final plasmid. In this case, diffdigester.uni-jena.de suggested, among others, a restriction digest with XmnI. Created in BioRender. Reuter, S. (2025) https://BioRender.com/w2imgdp.

We used three typical examples from our wet lab to validate diffdigester.uni-jena.de. In the first example (Fig. 2), we replaced mTFP (monomeric teal fluorescent protein) with a T2A-CD14 (glycoprotein recognition protein) cassette. Since not all restriction enzymes produced compatible overhangs, we used an adapter oligonucleotide. In the second example (Fig. 3), we replaced a gene for eGFP (enhanced green fluorescent protein) by tdTomato, another fluorescent protein. Here, we did not need to use an adapter oligonucleotide. In the third example (Fig. 4), we inserted DNA oligonucleotide encoding the target-specific sequence of the small guide RNA (sgRNA) that is used by the nuclease Cas9 to modify the human IL6 gene in this case. Here, the recipient plasmid and the final plasmid only differ by a few base pairs, which makes it more difficult to find a suitable enzyme. In all three examples, we could quickly find a suitable enzyme with diffdigester.uni-jena.de. The actual restriction patterns match very well the patterns predicted by diffdigester.uni-jena.de. However, the resolution by diffdigester.uni-jena.de is higher; examples 1 and 3 have two thick bands, which are actually two, and example 1 has also two bands close together. The real electrophoresis gels only show one band for each there.

Even though we created diffdigester.uni-jena.de to find the optimal restriction enzymes for differential digests, we should note that it can be used for all applications that involve comparing any DNA molecules not only plasmids with restriction enzymes. This expands its use to other methods such as using restriction fragment length polymorphisms [13] to diagnose a certain genetic trait. Here, diffdigester.uni-jena.de can be used to find the enzyme that targets a specific diagnostic single nucleotide polymorphism. Diffdigester.uni-jena.de can also help to find the optimal enzyme for the fragment number identification method (FN-Identify) to distinguish different species of bacteria efficiently [14]. Furthermore, diffdigester.uni-jena.de accepts completely unrelated non-homologous sequences, and thus can be used to find the optimal enzyme to detect known possible contamination in plasmid stocks.

## Conclusion

With diffdigester.uni-jena.de, we provide a tool to quickly find the optimal enzyme for a differential digest. It predicts the restriction pattern, so that the identity of the nucleic acids under question can be efficiently determined in a digestion analysis.

## Data Availability

The diffdigester site is free to all users. It does not require registration, and is publicly available at https://diffdigester.uni-jena.de/. The source code is available at https://github.com/MartinGuehmann/diffdigester/ and https://doi.org/10.5281/zenodo.15207958.
